# Enhancing the Sensitivity of Lateral Flow Assay with Europium Nanoparticles for Accurate Human IgG Quantification

**DOI:** 10.3390/mi14111993

**Published:** 2023-10-27

**Authors:** Satheesh Natarajan, Aashish Priye

**Affiliations:** 1 Healthcare Technology Innovation Center, Indian Institute of Technology, Chennai 600113, India; satheesh@htic.iitm.ac.in; 2 Department of Chemical and Environmental Engineering, University of Cincinnati, Cincinnati, OH 45221, USA; 3 Digital Futures, University of Cincinnati, Cincinnati, OH 45221, USA

**Keywords:** lateral flow assay, immunoglobulin G, europium nanoparticles, sensitivity, accuracy, clinical diagnostics

## Abstract

Accurate quantification of immunoglobulin G (IgG) levels is vital for understanding immune status and diagnosing various medical conditions. Lateral flow assays (LFAs) offer rapid and convenient diagnostic tools, but their sensitivity has been a limitation. Our research introduces a refined method incorporating europium nanoparticles, enhancing both sensitivity and accuracy of LFAs in human IgG measurement. Utilizing a unique sandwich format, carboxylate-modified polystyrene Eu (III) chelate microparticles (CM-EUs) acted as the primary reporters. The concentrations of both detection and capture antibodies on the strip were optimized to bolster the LFA’s quantitative performance. The subsequent calibration curve between the IgG concentration and the measured intensity ratio (*V_R_*) established the linearity and analytical sensitivity of our method with a high correlation coefficient (r = 0.99) and an impressively low limit of detection (LoD = 0.04 ng/mL). Our precision assessment, segmented into intra-assay and inter-assay evaluations, showcases the method’s consistency and reproducibility. The LFA assay’s stability was established by demonstrating its resistance to degradation and affirming its potential for extended storage without a dip in performance. The study’s findings underscore the potential of this method to contribute to diagnostic medicine and improve patient care.

## 1. Introduction

In the realm of modern diagnostics and biomedical research, accurate measurement of specific biomolecules within biological samples stands as a fundamental pillar. Among these biomolecules, immunoglobulins (commonly known as antibodies) are vital as they are generated in response to foreign antigens (such as pathogens or toxins) and help mediate the immune system’s reactions to infections and physiological events. Immunoglobulin G (IgG) is the predominant antibody class in human serum, contributing to approximately 75% of total serum immunoglobulins and serving key roles in pathogen neutralization and immune regulation [1,2,3,4]. It is part of a broader family of immunoglobulins that play essential roles in both humoral and cellular immunity [5,6].

The quantification of IgG-subclass proteins and antibodies finds its most suitable technique in immunoassays. These assays come in various formats, including solid-phase, liquid-phase, competitive, and noncompetitive binding immunoassays [7]. Among these, the noncompetitive two-site immunometric assay, using monoclonal antibodies specific to human IgG subclasses, has gained prominence for its robustness and precision in quantifying IgG subclass proteins [8,9,10]. This methodology is significant in diagnosing selective and total IgG-subclass deficiencies, often resulting from inherited structural or regulatory gene abnormalities [11]. Traditionally, methods like enzyme-linked immunosorbent assay (ELISA) [12] and Western blotting [13] for the detection and quantification of IgG are typically conducted in a laboratory setting. Recently, lateral flow assays (LFAs) have emerged as an alternative, offering rapid results. They do not require specialized equipment or extensive training, making them more suitable for point-of-care testing [14,15,16]. Lateral flow assays (LFAs) are paper-based tests utilizing capillary action to move a sample along a paper membrane. The mechanism involves the sample migrating through a pad containing labelled antibodies or antigens. If the target molecule (e.g., a specific IgG) is present, it binds to the labelled component pre-dried on paper, forming a complex that further migrates to a detection zone where it is captured, producing a visible line. LFAs are commonly used for screenings due to their speed and ease of use, often producing rapid results with minimal equipment needs. However, limitations encompass lower sensitivity and specificity compared to lab-based methods, and detection is typically qualitative or, at best, semi-quantitative. The integration of fluorescent reporters in LFAs provides more quantitative results but faces challenges such as low emission levels, colloidal instability, and chemical reactivity of complex colloids. These issues can compromise sensitivity and assay stability [17]. Concurrently, introducing dual or multiple detection zones, which target several antigens or antibodies simultaneously, has enhanced specificity. However, these strategies also present additional complexities in assay development and increase costs. Multiple detection zones, while increasing specificity, may complicate result interpretation and potentially increase the chances of non-specific binding. To overcome these challenges and further enhance the effectiveness of LFAs, utilizing europium chelate (Eu [III]) nanoparticles represents a significant advancement. Eu[III] nanoparticles offer enhanced sensitivity, quantitative capabilities, and a longer fluorescence lifetime than traditional fluorophores, thus reducing background noise and improving measurement accuracy [18].

Incorporating nanoparticles, especially gold nanoparticles [19,20] and quantum dots [21], as labels has been instrumental in augmenting visibility and sensitivity. Nanoparticles enhance the sensitivity and accuracy of lateral flow assays (LFAs) through multiple mechanisms. Their high surface-area-to-volume ratio allows for more effective immobilization of bio-recognition elements, leading to improved capture efficiency. Additionally, their unique optical properties, such as localized surface plasmon resonance, amplify colorimetric signals for easier detection of low-abundance analytes. Uniform size and shape contribute to assay reproducibility, thereby increasing accuracy. Furthermore, nanoparticles permit the incorporation of advanced detection techniques like magnetic- or fluorescence-based methods, offering additional routes for performance optimization. Europium chelates (Eu[III]) nanoparticles have revolutionized immunoassay development by offering significantly enhanced sensitivity and quantitative capabilities when compared to traditional particles, such as colloidal gold. This improvement can be attributed to the unique properties of europium chelate complexes, including their longer fluorescence lifetime (in microseconds, µs) as opposed to traditional fluorophores (which typically have nanoseconds, ns). This longer fluorescence lifetime allows for the collection of signals beyond the background fluorescence’s lifetime [22]. Furthermore, europium chelates possess a long Stokes shift, meaning that incident light from the excitation source (typically at a wavelength of around 330–340 nm) does not interfere with the detection of emitted light (typically at a wavelength around 610–620 nm) [18]. These distinctive characteristics, in combination with the availability of compact and portable time-resolved fluorescence (TRF) readers, open up new opportunities in the advancement of rapid diagnostic assays.

In this study, we demonstrate an anti-human IgG kit that utilizes carboxylate-modified polystyrene Eu (III) chelate microparticles (CM-EUs) as reporters to enhance the signal generated from LFAs. These reporters are pivotal in developing a cutting-edge LFIA system for precisely detecting anti-human IgG in human serum. To quantify results effectively, we adopt intensity volume ratio (*V_R_*)—a key parameter to quantify the target concentration that involves the fluorescence volume of both the test and control lines. Our comprehensive evaluation of the proposed LFA system based on polystyrene Eu (III) chelate microparticles includes establishing the linearity in signal response, analytical sensitivity, reproducibility, accuracy, and cross-reactivity. Our experimental findings unequivocally demonstrate the potential of this methodology for quantitative analysis of anti-human IgG levels.

## 2. Materials and Methods

### 2.1. Reagents

Human IgG (I2511), anti-human IgG (I1886), anti-human IgG chain-γ-specific (A6029), chicken IgY (CIgY), bovine serum albumin (BSA), and goat anti-chicken IgY (anti-CIgY) were purchased from Sigma-Aldrich (St. Louis, MO, USA). Additionally, 4-morpholineethanesulfonic acid (MES), 1-ethyl-3-(3-dimethylaminopropyl) carbodiimide hydrochloride (EDC), a centrifugal filter unit with an Ultracel-50 membrane, and N-hydroxysulfosuccinimide (sulfo-NHS) were obtained from the same source. CM-EUs (200 nm) were procured from Thermo Fisher Scientific Inc. (Waltham, MA, USA). Sample pads, conjugate pads, nitrocellulose membranes, and absorbent pads sourced from Millipore (Bedford, MA, USA). Trehalose was obtained from SRL Chemicals (Chennai, India). The Automated Lateral Flow Reagent Dispenser (ALFRD) was purchased from Claremont BioSolutions (Upland, CA, USA).

Buffer solutions, including sample pre-treatment buffer (1 × PBS, 0.5% BSA, 0.1% T-20), conjugate pad pre-treatment buffer (1 × PBS, 0.5% BSA, 10% Sucrose, 0.1% T-20), activating buffer (25 mm MES, pH 6.1), binding buffer (25 mm PB), wash buffer (1 × PBS, 0.5% BSA, 0.1% T-20), and blocking buffer (25 mm PB, 2% BSA), were freshly prepared before use.

### 2.2. Preparation of CM-EUs Conjugation with Anti-Human IgG

To prepare CM-EUs conjugated with anti-human IgG, 2 mg of CM-EUs was suspended in an activating buffer containing 10 mm sulfo-NHS and 1.25 mm EDC for one hour. The mixture was then centrifuged at 15,000× *g* for 20 min at 8 °C to remove the supernatant. The activated CM-EUs were washed twice and resuspended in a binding buffer. Subsequently, 50 µg of anti-human IgG was added to the activated CM-EUs, and the mixture was vibrated for 2 h. Uncoupled conjugates were removed by centrifugation at 10,000× *g* for 15 min at 8 °C. After two washes, blocking buffer was added and shaken for 1 h. The supernatant was discarded, and the conjugates were centrifuged at 10,000× *g* for 15 min at 8 °C. This washing process was repeated thrice. Finally, the conjugates were resuspended in 0.2 mL of labelling antibody dilution buffer, resulting in a CM-EUs concentration of 10 g/L. The conjugates of CIgY and CM-EUs were prepared using the same method.

The CM-EU test strip construction encompassed a well-structured assembly of components, including a sample pad, conjugate pad, NC membrane, and absorbent pad. For this purpose, the conjugates of CM-EUs linked with anti-human IgG and those tethered with CIgY were meticulously diluted to concentrations of 0.4 ng/mL and 0.025 ng/mL, respectively. These judiciously formulated conjugates were then precisely dispensed onto the conjugate pad at a rate of 5 µL/cm, employing a Claremount dispenser. Subsequently, the laden pad was subjected to a gentle drying process at 37 °C for 2 h to ensure optimal conjugate immobilization.

The crucial next step involved the strategic application of anti-human IgG and anti-CIgY. Specifically, anti-human IgG, meticulously prepared at a concentration of 1 mg/mL, was strategically dispersed onto an NC membrane at a controlled rate of 1 μL/cm. In parallel, anti-CIgY, also thoughtfully prepared at a concentration of 1 mg/mL, was similarly applied on the NC membrane. These distinct entities were destined to become the test and control lines. The membrane was dried at 37 °C for 3 h to ensure their stability and integrity.

Precision was exercised in cutting the strip into 3 mm widths using a specialized strip cutter to finalize the creation of the test strip. The resulting strips were then carefully housed within strip cassettes, maintaining their structural integrity. It is worth noting that the prepared test strips were stored in a controlled drying oven until they were ready for use.

This comprehensive procedure ensured the meticulous construction and preservation of the CM-EUs-based test strip, setting the stage for accurate and reliable assay outcomes.

### 2.3. Calibration

Measurements were conducted in ng/mL units to establish accurate anti-human IgG levels. These measurements were meticulously correlated with the First International Standard (2013) [23] for human IgG, assigned a standardized unit of 50 ng/mL, as established by the National Institute for Biological Standards and Control (NIBSC), under the code 95/522. This internationally recognized anti-human IgG standard material, regarded as the gold standard within our laboratory, served as the reference against which our LFIA based on polystyrene Eu (III) chelate microparticles was calibrated. The calibration process involved serial dilution of this certified standard material, ensuring a calibration range characterized by a theoretical value-to-measured value ratio falling within the range of 0.9 to 1.1, signifying precision and accuracy.

### 2.4. Equation to Calculate the Volume Ratio

To calculate the volume ratio, we determine the pixel volume by summing all the pixels within a designated region defined by both test and control lines. In the case of the NC membrane, the test area is established, and the control lines maintain a consistent size. We mark the measurements of rows and columns for these lines, effectively creating rectangular boundaries for our region of interest (ROI).

The volumes of both the control and test lines are determined based on the values obtained from 2D pixel intensity quantification within their respective boundaries. Below are the equations for calculating the test volume (V_T_) and control volume (Vc).

$$V_{C}=\sum_{Control\;line}I(x,y)$$

(1) $$V_{T}=\sum_{Test\;line}I(x,y)$$

The volume ratio (*V_R_*) is the ratio of the test line volume (V_T_) to the control line (V_C_) volume:

*V_R_* = V_T_/V_C_ (2)

The result of the human IgG concentration is calculated by applying an assay-specific calibration function, such as a polynomial curve, linear line curve, 4-parameter logistic regression, or a power equation. The coefficient of variation (CoV) was calculated using the equation

CoV = SD/mean × 100 (3)

### 2.5. Statistical Analysis

The human IgG standard curve was constructed by plotting the logit-log against the logarithm of the corresponding human IgG concentrations (X). This involved defining the *V_R_* (volume ratio) ratios between the human IgG standard as Bo (baseline) and Bx (sample). To ensure the reliability of the results, signal linearity and correlations were scrutinized using Pearson’s linear regression equation.

McNemar’s test and Pearson’s correlation were executed for comprehensive data analysis using SPSS 13.0 (Chicago, IL, USA). A statistical significance threshold of *p* < 0.05 was applied. Furthermore, sample means and standard deviations (SD) were calculated precisely using Microsoft Excel (version 2.12; Analyse-it Ltd., Leeds, UK). This meticulous statistical approach ensured rigorous analysis, accuracy, and the generation of meaningful correlations, supporting the results’ robustness.

## 3. Results

### 3.1. Principle of the Assay Method

**Figure 1:**
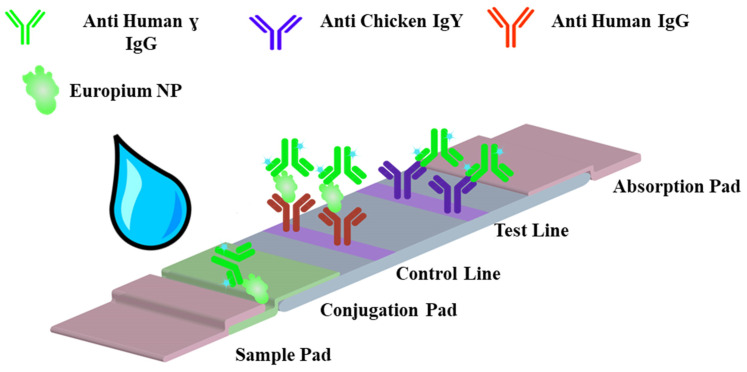
The schematic diagram of the quantitative lateral flow assay utilizing the europium nanoparticle. The blue drop represents the serum sample containing human IgG.

**Figure 2:**
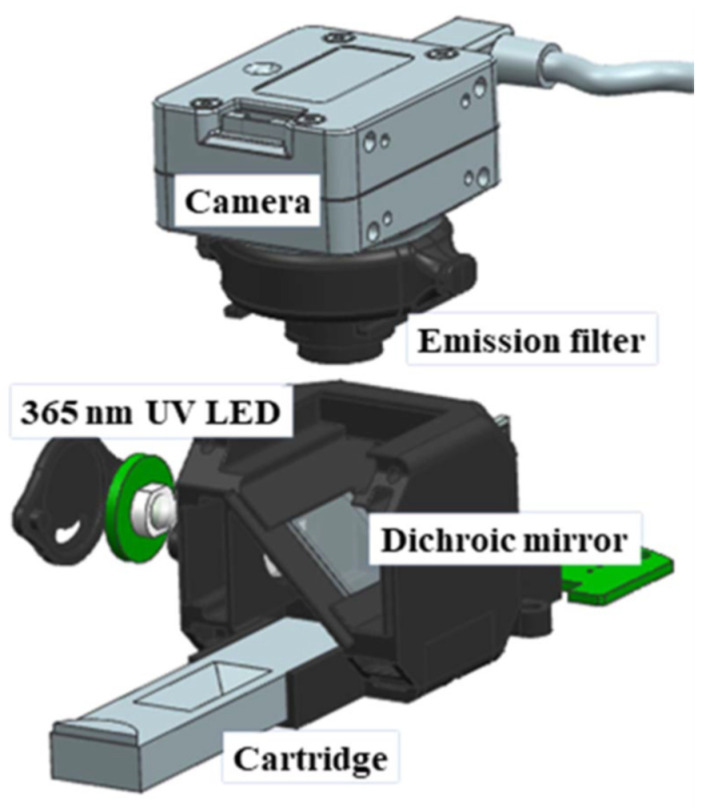
Exploded view of the developed immunoanalyzer and its parts, such as optical components, LED, and camera, were placed in a 3D-printed enclosure.

In lateral flow assays (LFAs), the primary working principle is capillary action that draws the sample along the strip, facilitating interactions between the analyte and the bio-recognition elements. The proposed LFA for quantifying human IgG levels follows the principles of a sandwich lateral flow format, as shown in Figure 1. To initiate the assay, a sample buffer containing human IgG is added to the sample pad. Utilizing the capillarity of the absorbent pad, the carboxylate-modified polystyrene Eu (III) chelate microparticles (CM-EUs) conjugated with anti-human γ IgG migrate across the nitrocellulose (NC) membrane and interact with the anti-human IgG on the test line. Simultaneously, CM-EUs conjugated with chicken IgY are captured by anti-chicken IgY on the control line, resulting in the appearance of a fluorescent band on both the test and control lines. This results in distinct fluorescent bands at both the test and control lines. Upon completion of the assay, the resultant test strip is assessed using a time-resolved fluorometry (TRF) immunoanalyzer (Figure 2) [24], which accurately measures the volume ratio (*V_R_*) between the test line and the control line (Figure 1). In the crux of this sandwich assay system, the CM-EUs conjugated with anti-human IgG create a ‘sandwich’ formation with the human IgG molecules present in the sample.

Notably, the fluorescence intensity exhibited on the test line is directly proportional to the concentration of human IgG within the sample. This correlation ensures that higher levels of human IgG yield more pronounced fluorescence signals. In stark contrast, the fluorescence intensity observed at the control line remains almost constant across varying human IgG concentrations. This steadfastness serves as an internal control mechanism, affirming the accurate migration of the components throughout the assay and the functional integrity of the overall process.

The precision of the method is primarily attributed to the use of the volume ratio (*V_R_*) for measurement of intensities of the test and control lines, which enhances accuracy and underscores its suitability for clinical applications. Our europium-nanoparticle-based immunoanalyzer adopts an optical setup comprising an excitation source, a camera, and fluorescence filters (Figure 2). When the strip is inserted into the TRF, a 365 nm ultraviolet-light-emitting diode (LED) illuminates the strip. The camera positioned directly above the test strip captures the emitted fluorescence signals. The incorporation of a wavelength-selective dichroic mirror and an emission-side filter ensures only the wavelength-shifted re-emitted fluorescence light reaches the camera, effectively filtering out the intense source light. This design permits high gain and prolonged exposure durations, enabling the detection of fluorescence from dilute concentrations. All optical elements, including the LED and camera, are housed within a 3D-printed enclosure.

### 3.2. Optimization of Europium Chelate LFA

**Figure 3:**
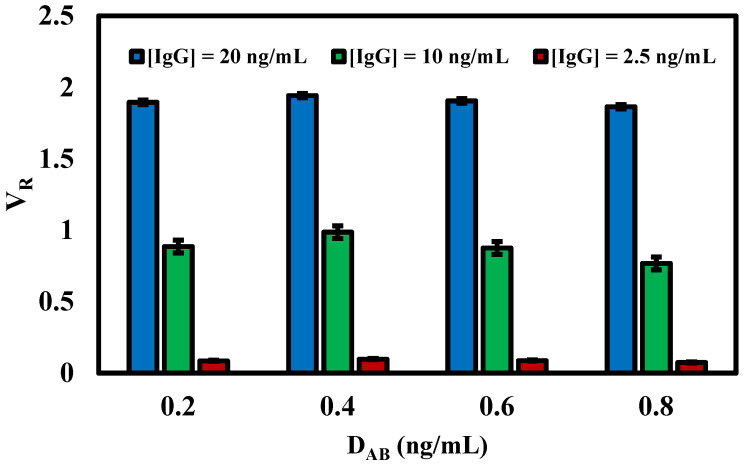
The optimal concentration of detection antibody (D_AB_) on the conjugation pad was evaluated by comparing volume ratio (*V_R_*) with varying amounts of the detection antibody coated on the conjugation pad (0.2, 0.4, 0.6, and 0.8 ng/mL) against the concentrations of the human IgG (20, 10, and 2.5 ng/mL). (*n* = 3).

**Figure 4:**
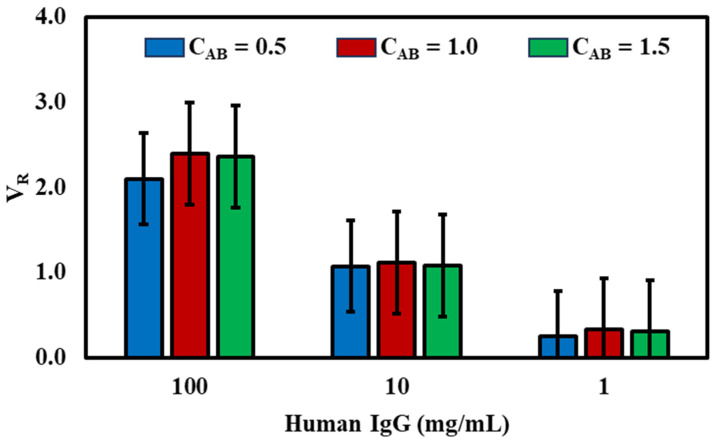
The optimal concentration of capture antibody (C_AB_) on the test line was evaluated by comparing volume ratio (*V_R_*) with varying amounts of the capture antibody coated on the test line against the three concentrations of the human IgG (1.5, 1.0, and 0.5 mg/mL). (*n* = 3).

To enhance the efficiency and sensitivity of our fluorescent lateral flow immunoassay, we systematically optimized various critical factors affecting assay performance. These factors included the quantity of CM-EUs (carboxylate-modified polystyrene europium chelate) conjugated with the detection antibody (goat anti-human IgG (γ-chain-specific)) on the conjugation pad and the concentration of the capture antibody (antibody–human IgG) on the test line. Our optimization process involved adjusting one parameter while keeping the others constant, as detailed in Table 1 and illustrated in Figure 3 and Figure 4.

### 3.3. Effect of Detection Antibody (D_AB_) Concentration on the Conjugation Pad

Determining the optimal concentration for antibody labeling can lead to improved sensitivity and specificity of the assay. If the signal intensity does not increase proportionally with the antibody amount, then an excess of antibodies could potentially lead to wastage or even reduce the accuracy of the assay. This study focused on the evaluation of four distinct concentrations of the detection antibody (D_AB_)–anti-human IgG (γ-chain-specific), labelled with CM-EUs: 0.2, 0.4, 0.6, and 0.8 ng/mL. To optimize the parameter, we consistently employed Human IgG concentrations of 2.5, 10, and 20 ng/mL throughout our experimental runs. A notable observation was the increasing volume ratio (*V_R_*) in tandem with the concentration of antibodies labelled with CM-EUs, peaking at 0.4 ng/mL, and subsequently showing a decline (Figure 3). This non-linear relationship between signal intensity and the antibody amount per CM-EUs suggests that there might be an optimal concentration range for antibody labelling, beyond which saturation or other inhibitory effects could be influencing the results. One potential explanation could be steric hindrance or competitive binding, where increasing concentrations of antibodies might prevent effective labelling or interaction with CM-EUs. Additionally, it’s possible that at higher concentrations, there might be aggregation of antibodies or CM-EUs, leading to decreased signal detection. This observation underscores the importance of meticulous calibration and optimization in assay development to ensure reliability and reproducibility. Consequently, we selected anti-human IgG (γ-chain-specific), labelled with CM-EUs at a concentration of 0.4 ng/mL as the optimal amount for each strip.

### 3.4. Impact of Capture Antibody (C_AB_) Quantity on the Test Line

We also examined the impact of the concentrations of the capture antibody (C_AB_) on the test line corresponding to C_AB_ = 0.5, 1.0, and 1.5 mg/mL. In Figure 4, we present the outcomes from detecting varied human IgG concentrations (1, 10, 100 ng/mL) under specific C_AB_ conditions. The data indicate that a C_AB_ concentration of 1 mg/mL is optimal, offering the highest sensitivity and detection range. This can be attributed to a balance between antibody availability and potential steric or competitive effects. At lower C_AB_ concentrations, there might be insufficient antibody to effectively capture and form complexes with the target human IgG. Conversely, at higher concentrations, there could be instances of antibody aggregation, reduced mobility, or even competitive binding, thereby compromising the assay’s sensitivity. Considering factors such as the volume ratio and cost-effectiveness, we determined that the optimum amount of capture antibody on the test line should be 1 mg/mL, used in subsequent assays.

The validation of the immunoassay kits developed is a comprehensive procedure to determine the kit’s appropriateness for its designated use. This validation aims to confirm the kit’s ability to produce consistent, accurate analytical data. For the human IgG LFIA kit, the validation involves a set of tests as outlined in the CDER guidance protocol [25]. The key parameters for method validation encompass linearity, analytical sensitivity, limit of detection, precision, and stability [26,27].

### 3.5. Linearity and Analytical Sensitivity

**Figure 5:**
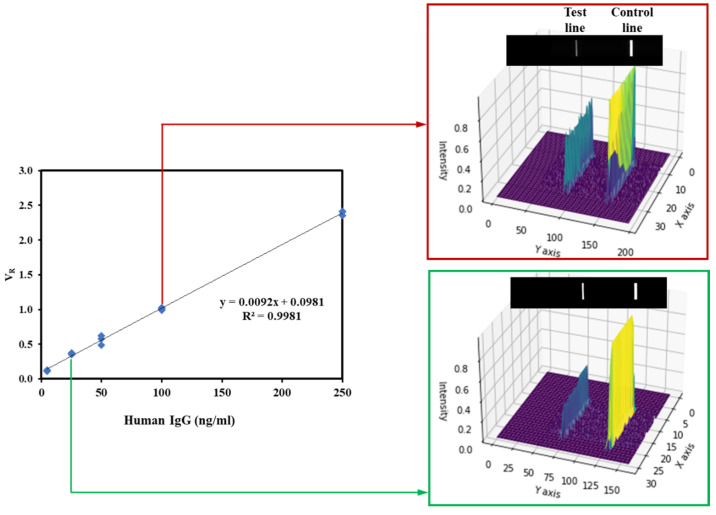
A calibration curve was constructed to detect human IgG using LFA cartridges. This involved utilizing an analytical strip made of nitrocellulose and utilizing Eu-labelled detection. The central parameter employed in this evaluation was the ratio of pixel volume between the test line and the control line, denoted as *V_R_* (*V_R_* = V_T_/V_C_). This ratio was plotted against the concentration of human IgG. The experiments were executed in triplicate, ensuring robustness, and the resultant data were subjected to fitting via linear regression.

The fundamental objective of this analysis was to discern the linearity and analytical sensitivity of the assay with respect to human IgG concentrations. To this end, a range of IgG concentrations (0, 50, 100, 150, 200 ng/mL) was chosen and accurately integrated within the sample buffer. Upon analysis using the europium-based lateral flow strip reader, the data reveal a definitive calibration curve (Figure 5). This curve was formulated by rigorously documenting the fluorescence intensities and juxtaposing the volume ratio (*V_R_*) with the respective human IgG concentrations. The obtained relationship is succinctly represented by the equation *V_R_* = 0.0092 × [*IgG*] + 0.0981 with a high correlation coefficient of r = 0.9981, which is indicative of the assay’s pronounced linearity and its ability to precisely quantify human IgG across the entire concentration range down to a concentration of 0.04 ng/mL. The high degree of linearity observed suggests minimal interference or anomalies in the assay, which can be attributed to the preparation of standards and the inherent efficiency of the europium-based lateral flow system.

In immunoassay test kits, terms like sensitivity, limit of detection (LoD), and limit of blank (LoB) denote the minimum detectable concentration of an analyte with significant reliability [28]. In accordance with these guidelines, we determined the LoB and LoD. The calculated LoB was 0.004 ng/mL, derived from the mean of blank values augmented by 1.645 times the standard deviation (n = 20). The LoD was set at 0.003 ng/mL, which is thrice the standard deviation of blank measurements (n = 20). These findings are summarized in Table 2. The high correlation coefficient combined with an extremely low LoD underscore the assay’s reliability and its potential utility in diverse applications where accuracy is paramount. 

### 3.6. Precision

Precision in immunoassays is categorized into intra-assay and inter-assay types. Intra-assay precision measures the consistency of results within a single experimental run, indicating short-term reliability. Inter-assay precision assesses variability across multiple runs, different operators, or reagent batches, providing insight into long-term robustness. Both types of precision are essential for ensuring the assay’s overall accuracy and reliability in diverse settings and are typically evaluated by measuring the same sample multiple times under varying conditions.

For the intra-assay evaluation, the experiment was executed within a singular day, encompassing ten replicates at each specified concentration: 0.5, 5, and 500 ng/mL. Contrastingly, the inter-assay was spread over three consecutive days, with each day witnessing three replicates at the aforementioned concentrations. The data generated were scrutinized by using one-way analysis of variance (ANOVA), and the results were summarized in Table 3. The inter-assay’s coefficient of variation spanned a range from 4.5% to 5.1%. Meanwhile, the intra-assay exhibited a more constricted range, from 4.7% to 4.5%. Such tight ranges in coefficient of variation values are indicative of the assay’s high degree of consistency and reproducibility. The statistical significance of the results, residing comfortably within the permissible thresholds, further bolsters the validity of the assay. These data, in conjunction with the observed coefficients of variation, justify the assay’s potential suitability for clinical analytical applications. 

### 3.7. Stability of the LFIA Kit

**Figure 6:**
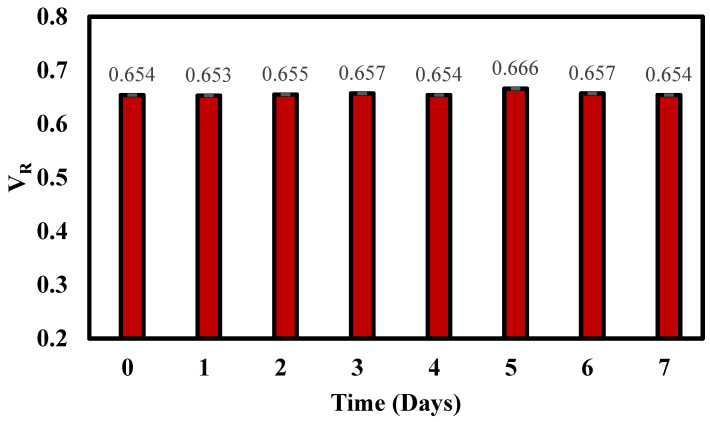
Evaluation of LFIA storage stability with accelerated ageing tests. The LFIA strips were stored at 37 °C in aluminum pouches with desiccant. Every single day for one week, three strips were removed from each set and used to analyze samples.

Another pivotal aspect of the LFIA kit’s utility is its stability as this dictates the permissible storage duration post-preparation before the kit is subjected to the final analysis. Establishing this parameter is quintessential to ensure that the analyte’s performance remains unscathed, leading to consistent and trustworthy outcomes. To determine the stability of the LFIA strips, an accelerated aging test was employed. For this, the strips were sequestered at a temperature of 37 °C, spanning a time frame of seven days. A noteworthy observation from this test was the volume ratio (*V_R_*) consistency, which showcased negligible variance throughout the accelerated aging test, even under the elevated temperature condition for the entire week (Figure 6). The consistency in *V_R_* values is indicative of the strip’s high resilience against potential degradation or alteration in performance due to prolonged storage. In essence, the strips appear to retain their efficacy and are likely to produce reproducible results even after being stored for extended periods under specified conditions.

## 4. Conclusions

Our research evaluates the quantitative performance of a europium-based lateral flow assay (LFA) designed for the precise quantification of human IgG levels. This assay combines a distinct architecture with an analytical strip formed of nitrocellulose and Eu-labelled detection to enable a robust and sensitive platform for accurately determining human IgG concentrations. The concentrations of both detection and capture antibodies on the strip were optimized to enhance the LFA strip’s performance. The subsequent calibration curve between the IgG concentration in the sample and the measured intensity ratio (*V_R_*) establishes the linearity and analytical sensitivity of our method, presenting a high correlation coefficient (r = 0.99) and an extremely low limit of detection (LoD = 0.04 ng/mL). Our precision assessment, bifurcated into intra-assay and inter-assay evaluations, underlines the method’s remarkable consistency and reproducibility. The observed coefficients of variation, consistently narrow across both types of precision tests, further reinforce the assay’s potential clinical applicability. The uniformity in i values, despite a week-long storage at 37 °C, attests to the strip’s robustness against degradation, emphasizing its potential for prolonged storage without compromising on efficacy.

While our findings underscore the potential of the europium-based lateral flow assay (LFA) method in quantifying human IgG levels, they also highlight several challenges inherent to the study and the broader field of LFA. Real-world patient samples may introduce variances due to factors like sample matrix effects, concomitant medications, or disease states. Future studies should seek to adapt and validate the assay for a diverse range of analytes and biomarkers beyond human IgG, including real patient samples to establish its broader clinical applicability. This advancement contributes to the arsenal of diagnostic tools available to healthcare professionals, with promising implications for disease monitoring, diagnosis, and patient care.

## Figures and Tables

**Table 1:** Optimization of LFA concentration parameters.

Type of Parameters	Parameters Tested	Selected Parameter
Concentration of detection antibody in the conjugation pad	0.2, 0.4, 0.6, 0.8 ng/mL	0.4 ng/mL
Concentration of capture antibody in the test line	0.5, 1.0, 1.5 mg/mL	1 mg/mL

**Table 2:** Summary of the linear fitting and limit of detection parameters for the europium-based lateral flow assay.

Parameter	Eu-LFA
slope	0.0092
intercept	0.0981
R	0.9981
LOD (ng/mL)	0.004
LoB (ng/mL)	0.003

**Table 3:** Inter-assay and intra-assay variability for the human IgG lateral flow assay.

	Inter-Assay		
Human IgG (ng/mL)	Mean	SD	cv
0.5	10.8	6.0	4.5
5	35.9	1.7	4.9
500	131.8	0.5	5.1
	Intra-Assay		
Human IgG (ng/mL)	Mean	SD	cv
0.5	12.7	5.2	4.7
5	37.5	3.2	5.2
500	137.2	0.7	4.5

## Data Availability

Data can be provided upon request.
